# Papain Injection Creates a Nucleotomy-like Cavity for Testing Gels in Intervertebral Discs

**DOI:** 10.3390/gels10090571

**Published:** 2024-09-02

**Authors:** Jan Ulrich Jansen, Graciosa Quelhas Teixeira, Andrea Vernengo, Sybille Grad, Cornelia Neidlinger-Wilke, Hans-Joachim Wilke

**Affiliations:** 1 Institute of Orthopaedic Research and Biomechanics, Centre for Trauma Research Ulm, Ulm University, 89081 Ulm, Germanycornelia.neidlinger-wilke@uni-ulm.de (C.N.-W.); 2 AO Research Institute Davos, 7270 Davos, Switzerland

**Keywords:** chemonucleolysis, papain, range of motion, biomechanics, organ culture, disc height, nucleus replacement

## Abstract

Biomaterials, such as hydrogels, have an increasingly important role in the development of regenerative approaches for the intervertebral disc. Since animal models usually resist biomaterial injection due to high intradiscal pressure, preclinical testing of the biomechanical performance of biomaterials after implantation remains difficult. Papain reduces the intradiscal pressure, creates cavities within the disc, and allows for biomaterial injections. But papain digestion needs time, and cadaver experiments that are limited to 24 h for measuring range of motion (ROM) cannot not be combined with papain digestion just yet. In this study, we successfully demonstrate a new organ culture approach, facilitating papain digestion to create cavities in the disc and the testing of ROM, neutral zone (NZ), and disc height. Papain treatment increased the ROM by up to 109.5%, extended NZ by up to 210.9%, and decreased disc height by 1.96 ± 0.74 mm. A median volume of 0.73 mL hydrogel could be injected after papain treatment, and histology revealed a strong loss of proteoglycans in the remaining nucleus tissue. Papain has the same biomechanical effects as known from nucleotomies or herniations and thus creates a disc model to study such pathologies in vitro. This new model can now be used to test the performance of biomaterials.

## 1. Introduction

Lower back pain represents the leading reason for years lived with disability [1,2,3], and progressive degenerative changes are considered to be one of the major causes of spinal disorders [4,5]. Current treatments, i.e., conservative, surgical, or analgesic, are temporary solutions that often result in limited patient mobility or the degeneration of adjacent segments [6,7,8,9,10] and thus the demand for novel advanced motion-preserving treatment methods, e.g., the use of nucleus replacement implants [11] or strategies to regenerate the intervertebral disc (IVD) [12]. For this purpose, hydrogels injected into the IVD are often considered as a carrier material for cells or an independent therapeutic option [13,14,15,16,17] since they are already in clinical use for other application like the cornea as well as various musculoskeletal defects [13,18]. An increasing number of biomaterials to be injected into the IVD are showing promising results, such that preclinical in vitro and in vivo studies are required as the next step to determine the extrusion risk and biomechanical performance of the new materials [14,19,20]. Bovine models with fresh IVDs of the tail or the lumbar spine have become a well-accepted explant model in several studies due to their similarity to human lumbar disc regarding, e.g., cellular activity or tissue composition, but also because they are easy and ethically safe to obtain [19,21,22,23]. Furthermore, it has recently been demonstrated that models where the gel is applied through an intact annulus fibrosus (AF) maintain their biomechanical performance without an extrusion risk [24], whereas for models with an AF defect, closure of the AF is essential [25,26]. However, fresh bovine IVDs from young, slaughtered animals grant an intact AF, but injections with a needle and syringe of any substance into these IVDs are difficult and only small amounts can be injected. Because fresh IVDs from young animals do not show any signs of degeneration, they are fully filled with an intrinsic pressure and have no cavities, clefts, or fissures. Therefore, injectable volumes are relatively low. For example, according to a few studies, the injectable volume for fresh bovine lumbar IVDs is approximately 0.27–0.53 mL [24] and approximately 0.1–0.2 mL for fresh bovine tail IVDs [27,28,29,30,31]. Injected liquids may not even remain in the nucleus pulposus (NP) [30,32].

Previous studies searching for ways to artificially degenerate fresh, non-degenerated IVDs and enable injection have rediscovered chemonucleolysis—an alternative therapy for soft disc herniations that was already used in the 1980s and 1990s for treatment [28,33,34,35]. During chemonucleolysis, enzymes such as chymopapain are injected into the NP in order to degrade the proteoglycans, reducing the fixed charge density of the NP tissue, leading to the release of firmly bound water, resulting in a reduction in the intradiscal pressure and thus depressurizing the compression of the nerve route, with the goal to prevent further protrusions or herniations [35,36,37]. Chymopapain (EC 3.4.22.6) is one of the three main different proteinases present in papaya latex, apart from papain and papaya proteinase III [36,38] and was first experimentally injected into rabbit IVDs by Smith et al. in 1963 [39]. In clinical trials, chemonucleolysis has demonstrated a mean success rate of 74 ± 11% in 31 studies between 1971 and 1994 [33,34,35,40,41,42,43], but clinical use was discontinued due to dangerous complications such as anaphylactic shock and allergic responses, as well as controversies regarding efficacy [43,44,45,46,47].

Early studies, between 1975 and 1985, have examined the biomechanical effects of chymopapain, such as stiffness, disc height, histology, and ROM, whereby enzymatic digestion was performed in vivo in canine or human spines. Bitz and Ford radiologically measured a reduction in disc height of 8.4% in patients successfully treated with chymopapain for less than 2 months [48]. Canine in vivo experiments by Bradford et al. and Spencer et al. revealed that the stiffness of the chymopapain-treated motion segments decreased significantly under axial load and under torsion but that these changes regressed after three months [49,50]. After three months, the histological differences became smaller according to Bradford et al. [49], but Spencer et al. state that the long-time effects of chymopapain in vivo remain due to fibrotic changes in the NP [50]. Kahanovitz et al. used canine IVDs to compare the effect of chymopapain with the effect of discectomy produced by mechanical removal of the NP and of the inner AF fragments [51]. No significant differences were observed in disc height after chemonucleolysis and discectomy, but chemonucleolysis, also affecting AF, resulted in more severe instability. This means decreased tension of the annulus fiber layers and more disc bulging than after discectomy [51,52]. Dolan et al. reported first about injecting chymopapain into human cadaver IVDs ex vivo in order to understand the short-time effects after injection but could not find any effects on disc height, compressive stiffness, or flexibility in flexion [53]. With the first study by Roberts et al., research has shifted to using papain (EC 3.4.22.2), which is the minor constituent in the papaya latex (about 8%), easier to purify [28,38], available to purchase, and in the same range of specificity and activity as chymopapain [36]. Roberts et al., Chan et al., and others have started to use papain in several organ culture studies to mimic disc degeneration in animal IVDs and have reached a cavity formation within a time frame of 6–21 days [27,28,29,54,55,56,57]. Frequently, cavities have extended to the cartilaginous endplate and the AF. Some of the organ culture studies have reported biomechanical data such as disc height [57] or stiffness via compression testing [29,56,57], but neither the ROM of bovine tail functional spinal units nor the effects from cavities induced by papain have been measured to date.

In particular, the following conflicting requirements for such an experiment complicate the measurement of such values: Namely, cadaver specimens commonly used for in vitro experiments are limited to a 24 h period according to recommendations to avoid undesired post-mortem change and, similar to Dolan et al., creating cavities in the NP within a short time frame could not be achieved so far [53,58]. The aim of this experimental study was to develop an in vitro protocol with papain to create cavities in bovine tail segments, facilitating biomechanical testing. Subsequently, the study aimed to evaluate the effects of papain digestion on the ROM, the neutral zone (NZ), the disc height, the injectable volume, histological features, and the matrix composition.

## 2. Results and Discussion

A new cavity model with papain-treated bovine tail segments under seven days of organ culture and subsequent complex loading was developed, which allowed us to create cavities in the NP and to measure their effects on the bovine tail segments at different time points. Determined from the X-rays of 12 IVDs, the average disc diameter was 28 ± 1 mm and the average disc height at the anterior and posterior borders 15 ± 1 mm. The embedding of the cranial and caudal vertebral bodies in poly(methyl methacrylate) (PMMA) allowed the fixation in testing machines and, by using Penicillin-Streptomycin, Amphotericin B, and disinfection, no contamination became visible during the whole experiment. The bone–PMMA interface remained stable even after incubation in the cell culture medium. Final dissection of the functional spinal units of both groups, papain and sham, did not show any breakdown of the bone structure, loosening, or decay.

### 2.1. Papain Injection and Macroscopic Effects

**Figure 1:**
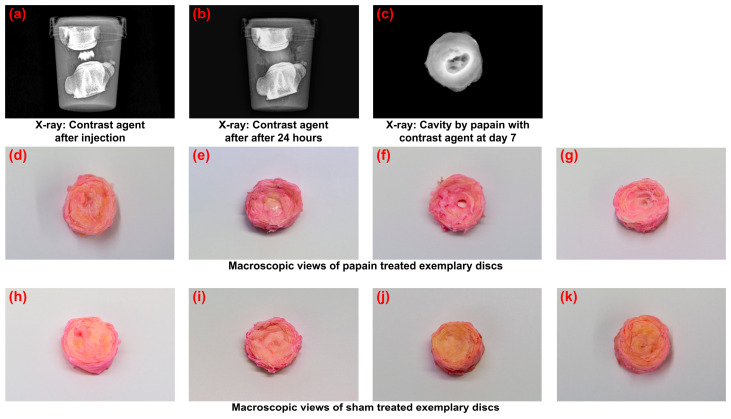
Papain injection and its macroscopic effects. (**a**,**b**) Embedded bovine tail functional spinal units in cell culture medium in sterile container. (**a**) shows papain with contrast agent after injection in the intervertebral disc (IVD) center and (**b**) shows state after 24 h incubation at 37 °C. (**c**) Transversal X-ray of cavity 7 days after the papain injection. (**d**–**g**) Transversal views of dissected discs on day 7 for papain group. (**h**–**k**) Transversal views of dissected discs on day 7 for sham group.

For all groups, papain, phosphate-buffered saline (PBS), and a contrast agent could be injected in a standardized manner up to 200 µL. No leakage occurred. When the contrast agent was injected into fresh discs and subsequently incubated, the clearly localized contrast agent in the NP disappeared and a shadow became visible in the entire bovine tail disc within 24 h (Figure 1a,b). On day seven, all dissected IVDs of the papain group exhibited a clear cavity in the center of the disc (Figure 1c–g). Some papain-digested discs also showed changes at the cartilaginous endplate. Fissures or clefts could not be detected macroscopically. Discs of the sham group did not exhibit any cavities or macroscopically visible defects (Figure 1h–k).

### 2.2. Flexibility

**Figure 2:**
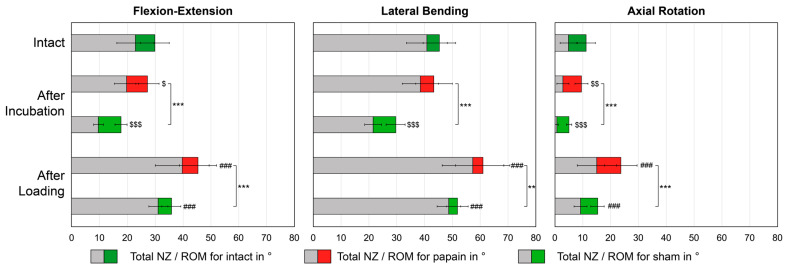
Flexibility. Mean range of motion (ROM) and mean neutral zone (NZ) with standard deviation in three motion planes for the intact condition (n = 36) and subsequent time points after−incubation and after−loading for specimens either treated with papain (n = 21) or phosphate-buffered saline (PBS) as sham (n = 15). Significant differences between the intact and the after−incubation state: ^$^ *p* < 0.05, ^$$^ *p* < 0.01, ^$$$^ *p* < 0.001. Significant differences between the after−incubation and the after−loading state: ^###^ *p* < 0.001. Significant differences between the groups for each time point; ** *p* < 0.01, *** *p* < 0.001.

The Shapiro–Wilk test confirmed the normality assumption in all subgroups of ROM and NZ; therefore, parametric tests were used. However, variance homogeneity of the data (all subgroups papain and sham) was not found by Levene’s test (*p* ≤ 0.021). The total ROM of the fresh bovine tail segments of CY34 averaged to 29.9 ± 5.2° for flexion–extension (FE), 45.4 ± 5.9° for lateral bending (LB), and 11.3 ± 3.3° for axial rotation (AR) in the intact condition (*n* = 36). The mean NZ were 22.9 ± 6.7°, 40.8 ± 7.3°, and 4.9 ± 3.0°, respectively. The highest ROM and NZ per group and condition were measured in the LB plane followed by FB and AR (Figure 2). For both groups, the ROM decreased significantly after incubation (d ≥ 0.59, *p* ≤ 0.004, n = 21/15), except for papain in the LB plane (d = 0.35, *p* = 0.110, n = 21). The NZ also decreased correspondently to ROM (d ≥ 0.52, *p* ≤ 0.013, n = 21/15), except for papain in the LB (d = 0.34, *p* = 0.119, n = 21). After loading, ROM and NZ increased significantly compared to the after-incubation state for all motion planes (d ≥ 0.91, *p* < 0.001, n = 21/15). After incubation, the ROM and NZ decreased significantly more for the sham discs than for the papain discs (d ≥ 1.31, *p* < 0.001, n = 21/15). Similarly, after complex loading, the ROM and NZ increased significantly less for the sham discs than for the papain discs (d ≥ 0.97, *p* ≤ 0.003, n = 21/15). The effect sizes of > 0.5 correspond to a strong effect. Throughout the whole protocol from intact to after loading, FE increased by +51.6% for ROM and by +73.3% for NZ with papain, whereas it increased by +19.8% for ROM and by +36.0% for NZ within the sham group. For LB, ROM increased by +34.3% and NZ by +40.6% for papain, whereas it increased by +14.1% and +19.6% for sham, respectively. The strongest overall increase was found in the AR plane, where for papain, ROM increased by +109.5% and NZ increased by +210.9%. For sham, ROM and NZ in the AR plane changed by +35.8% and +90.3%.

### 2.3. Disc Height

**Figure 3:**
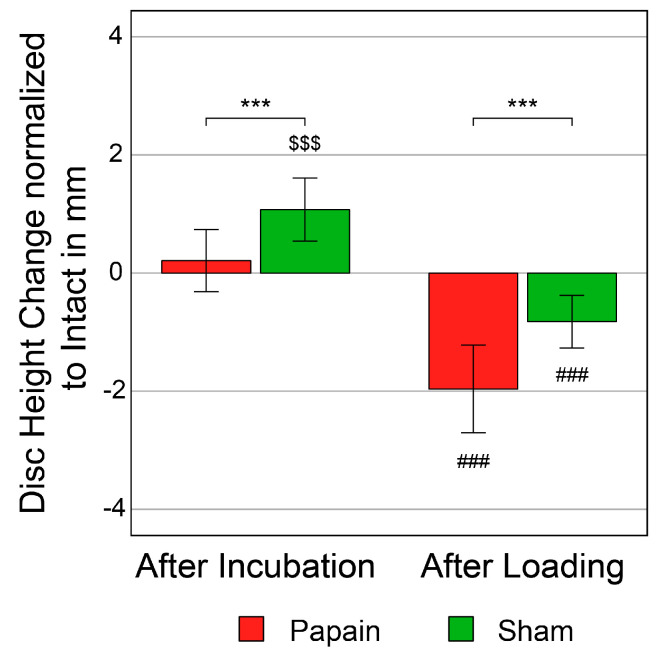
Disc height change. Plots of mean disc height change with standard deviation in mm for the time points after−incubation and after−loading normalized to the intact condition for specimens either treated with papain (n = 21) or PBS as sham (n = 15). Significant differences between the intact and the after−incubation states: ^$$$^ *p* < 0.001. Significant differences between the after−incubation and the after−loading states: ^###^ *p* < 0.001. Significant differences between the groups for each time point: *** *p* < 0.001.

The Shapiro–Wilk test detected no deviations from the normal distribution for the disc height, and variance homogeneity is achieved according to Levene’s test (*p* ≥ 0.127). In an opposite way to ROM and NZ, the disc height increased after incubation for both groups (Figure 3). This effect was significant for sham (d = 0.90, *p* < 0.001, n = 15) but not for papain (d = 0.37, *p* = 0.088, n = 21). After complex loading, the disc height significantly decreased in both groups (d ≥ 0.97, *p* < 0.001, n = 21/15). After incubation, the disc height significantly increased more for sham than for papain (d = 0.53, *p* < 0.001, n = 21/15) and, after complex loading, the height decreased significantly more for papain than for sham (d = 0.64, *p* < 0.001, n = 21/15). The effect sizes of >0.5 correspond to a strong effect. The strongest overall change in the disc height was determined for papain after loading with 1.96 ± 0.74 mm compared to the intact condition.

### 2.4. Injectable Volume

**Figure 4:**
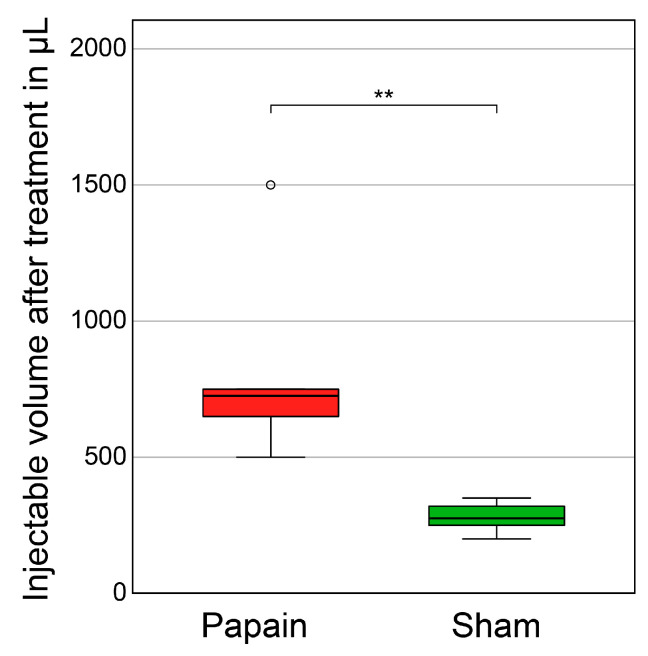
Injectable volume after treatment. Boxplots of injectable volume of hydrogel after incubation and loading in µL for specimens either treated with papain (n = 6) or PBS as sham (n = 6). Asterisks indicate significant differences between the groups. Level of significance: ** *p* < 0.01. Outliers: °.

Due to the sample size, the injectable volume was evaluated non-parametrically. IVDs of the papain group allowed significantly more volume to be injected than in the sham group (*p* = 0.002) (Figure 4). The median (range) in mL for papain was 0.73 (0.50, 1.50) and 0.28 (0.20, 0.35) for sham, respectively.

### 2.5. Histology and Proteoglycan Content Quantification

**Figure 5:**
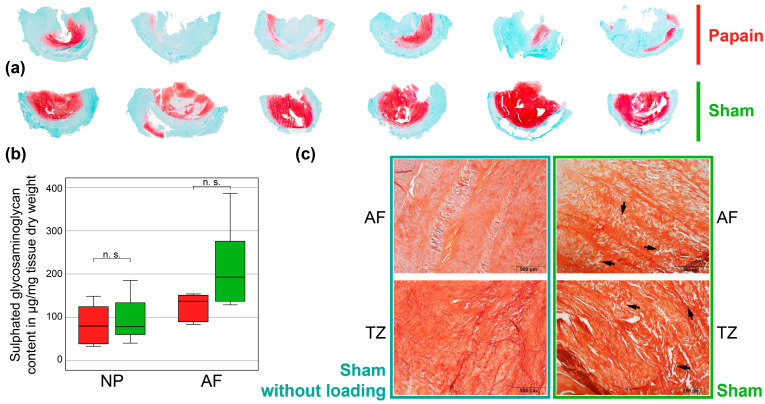
Effects of papain digestion and complex loading on matrix composition and tissue integrity. (**a**) Histological overview of representative half IVDs of the bovine tail (n = 6) for papain and sham. Safranin-O/Fast-Green colors proteoglycan-rich tissue red and other tissue, such as collagen, green. (**b**) Sulphated glycosaminoglycan content quantified at the end of the experiment on day 7 for papain and sham group. n = 6 tissue punches were taken from the nucleus pulposus (NP) and anulus fibrosus (AF). The sulphated glycosaminoglycan content was normalized to the dry weight of the punched tissue. (**c**) Histological staining of sham discs (left column) without complex loading versus sham discs (right column) with complex loading on day 7. Sagittal IVD sections were stained with Safranin-O/Fast-Green for visualization of the tissue. The upper row shows tissue from the AF whereas the lower row shows tissue from the transition zone (TZ) (scale bar, 500 µm). Arrows indicate exemplary micro-damage within the tissue.

After testing on day seven, six discs per group were further analyzed with staining and sulphated glycosaminoglycan quantification. One additional IVD without dynamic loading was also stained. With a red stain, Safranin-O staining revealed a strong decrease in proteoglycans within the NP for the papain compared to the sham discs (Figure 5a). Slight proteoglycan residues still seemed to be present in the transition zone to the AF, indicating a radial diffusion and activity of the enzyme from the injection point. To some extent, papain led to tissue loss in the NP. High magnification images of the AF and transition zone compare the effect of complex loading on the tissue integrity of exemplary discs of the sham group with and without loading (Figure 5c). The microscopic sections show small micro-cracks and delamination in the tissue with dynamic loading, which did not occur in the tissue without loading. The biochemical quantification determined significant higher glycosaminoglycan content for the AF than for the NP for both groups together (*p* = 0.003, n = 12) (Figure 5b). In the NP and AF, no significant differences between the groups occurred (*p* ≥ 0.126). In the AF, the glycosaminoglycans slightly decreased more for the papain than the sham treatment, at about 25%.

### 2.6. Discussion

Preclinical models are of high relevance to test the novel therapies for injection into the intervertebral disc. This study has successfully developed a new in vitro model with cavities for future performance tests of injected biomaterials such as gels. This study measured the biomechanical behavior of bovine tail segments after papain treatment, including the ROM, NZ, and disc height. The biomechanical results of existing in vivo studies from before 1985 in dogs and rabbits have been confirmed. The major changes after papain treatment are characterized by ROM and NZ increase, decrease in disc height, proteoglycan loss, and cavities in the disc. Furthermore, important findings for future in vitro experiments with biomaterial testing have been obtained. In particular, papain digestion allows for the easier injection of fluids and hydrogels into the IVDs and, after papain treatment, 2.6 times more hydrogel can be injected into these discs in comparison with the discs of the sham group. These injected hydrogels with or without cells are then considered as a future promising strategy to improve the biomechanical function of the NP and to have a regenerative effect on the disc [14,59,60]. Thus, the testing platform presented here represents an important contribution to the development of regenerative disc therapies. Furthermore, the papain model developed here has a similar effect as a nucleotomy on the IVD and can serve as a model for this purpose.

Fresh bovine tail discs are a well-accepted, widely used model for biomechanical studies and organ culture experiments. However, since IVDs from young animals usually do not show degenerative changes, they have strong limitations when used for the biomechanical testing of many clinical scenarios, e.g., regenerative treatments for degenerated discs [19,61,62]. Hence, chemonucleolysis with papain for artificial degeneration has been introduced by other research groups, but biomechanical effects such as the ROM of these models have not been investigated yet [28,29,54]. In the present study, biomechanical tests (ROM/NZ) with papain treatment in organ culture were made possible. By using organ culture of fresh tissue from the slaughterhouse, the new approach overcomes the problems with the usage of cadaver specimens, where it is limited to a test duration of 24 h in the defrosted state and enzymes such as papain must act for a longer period of time and at higher temperatures to create a cavity [53,58,63].

This study used a papain concentration of 65 U/mL (200 µL with 13 U per disc/1 mg lyophilized papain per disc) to create large, macroscopically visible cavities and even structural defects extending to the cartilaginous endplate within the nucleus of papain digested discs. No cavities occurred in the sham group as expected. Compared to the clinical use of papain, this concentration was comparatively low since chymopapain concentrations typically used in patients have ranged between 500–6000 U [34,47] or 2–8 mg [40,41] per disc. But in comparison to Roberts et al., Chan et al., and others who achieved cavity formation with 30–150 U/mL of papain and a volume of 70–200 µL/disc within a time frame of 6–21 days, this study has followed the current research practices [27,28,29,54,55,56,57].

For the first time, to our knowledge, ROM and NZ have been measured after in vitro treatment with papain. In this study, changes in ROM, NZ, and disc height occurred in two conditions—after incubation and after complex loading—with and without the presence of papain. In all cases, an inverse relationship was found, namely that a reduction in the ROM was associated with an increase in the disc height and a greater ROM with a decrease in the disc height. After incubation, the increase in disc height and the stiffening effect can be attributed to the fluid uptake of the disc as already widely studied [64,65,66,67,68,69,70]. As described by Yang and O’Connell, the free swelling of the disc increased the disc height and re-oriented the AF fiber closer to the axial direction [65]. This led hypothetically to increased tension in the AF and less mobility, i.e., less ROM, in all motion planes. When comparing the papain and the sham group after incubation, the significantly lower decrease in ROM and the significantly lower increase in disc height for papain are striking. This effect can be clearly attributed to the effect of papain, since the papain and sham group discs have undergone the same incubation parameters and the same biomechanical testing including complex loading. In chemonucleolysis, the proteoglycans in the disc tissue are broken down stepwise by enzymatic processes. The loss of proteoglycans then reduces the fixed charge density, and, for papain, the disc tissue can no longer bind the water as strongly as in the sham discs, leading to less height increase for papain-treated discs than for sham-treated discs after incubation. The effect of papain, such as reduced water-binding capacity and tissue weakening, is also evident in complex loading. The disc height decreases significantly more compared to the sham group and flexibility (represented by ROM) and stability (represented by NZ) of the motion segments decrease significantly, when treated with papain. This confirms the destabilizing effect that was already discovered for the clinical use of the chemonucleolysis before it was discontinued as patient treatment [49,50,51]. Additionally, enzymatic degradation of the proteoglycans is also clearly shown by the stained sections with Safranin-O (Figure 5a); compared to the sham discs, almost no red staining can be detected in the papain-treated discs, suggesting the dissolution and disappearance of the proteoglycan side chains.

For the ROM and NZ, the present study found similar values as those that Lu et al. had obtained for in vivo chymopapain-treated, lumbar canine discs after 1 week. For the ROM, an increase of 70.4%, 54.5%, and 127.8% is reported for FE, LB, and AR, respectively (values recalculated), whereas we measured 51.6%, 34.3%, and 109.5% [71]. For NZ, Lue et al. figured out higher values with increases of 243.8% for FE, followed by 350% for FB and 300% for AR compared to our study, which were 73.3%, 40.6%, and 210.9%. Within in vivo canine studies with chymopapain, Bradford et al. reported a remarkably significant decrease in stiffness from 876 ± 167 Nm/deg to 587 ± 175 Nm/deg for torsion, while Kahanovitz et al. measured 50% less torsional stiffness after six weeks [49,51]. In line with our study, papain is often associated with a particularly strong effect on the ROM in the AR motion plane [49,50,51,71]. This is explained by the hypothesis that torsional stiffness is particularly influenced by the AF and that its fibers are subjected to more shear stress due to papain weakening, which then leads to higher AR or a lower torsional stiffness [50]. The presence and effect of (chymo)papain in the AF is also proven by the loss of proteoglycans in the AF detected in various studies after papain or chymopapain treatment [28,29,50,54,56,71]. This is also supported by our data, which show a progression of papain enzyme into the AF in the histological staining (Figure 5a) and the distribution of the papain buffer into the AF after 24 h (Figure 1b). As first noted by Kahanovitz et al., the height decrease and inverse ROM increase by papain is very similar to the biomechanical behavior after a nucleotomy, which here describes the removal of NP tissue, and is widely documented in the literature [51,72,73,74,75]. Wilke et al. used a nucleotomy in a lumbar calf disc to test the impact of a collagen scaffold on the disc height and flexibility. In their study, the nucleotomy led to an increase in the total ROM of 27.9% in flexion–extension and a decrease in disc height of 0.84 mm [73]. Another study, by Heuer et al., showed, for human lumbar IVDs, that a nucleotomy also resulted in an increase in the ROM of flexion–extension of 263.8% for 7.5 Nm and a decrease in the disc height of 1.97 mm [75]. Human disc herniations, resulting in a loss of nucleus material, also cause an increased ROM and decreased disc height [51,74]. Hence, papain-treated discs with a large cavity could very well serve as a model for discs with nucleotomy or after herniation.

Due to a small injury to the AF by the 30 G needle (diameter 0.3 mm) in this bovine tail model, it does not fully reflect the clinical pathology where larger defects occur after a nucleotomy or herniation at the AF, but this distinction also offers many advantages for in vitro testing. Standardized defects in the AF could be subsequently placed, or the intrinsically closed disc created by papain could serve as a model where the disc is first filled with a biomaterial and then imaginarily closed by a perfect anulus closure implant. This would allow for tests where only the impact of a biomaterial within the NP is tested and not the AF integrity. AF injury became a controversial topic after Carragee et al. showed that discography, i.e., disc injection, promotes disc degeneration, and that new disc regeneration methods are mostly intended to be injected into the disc at later clinical applications [56,76]. Carragee et al. related their study to needle sizes of 22–25 G, but Elliott et al. have described that the needle-size-to-disc-height ratio plays an important role and that a smaller 30 G injury has no effect like NP depressurization in bovine tail discs [76,77]. Hence, for injecting papain or PBS, a needle size of 30 G was used in the present study. In the development, but also in the later clinical application of new injectable therapies, it is also of interest to inject a certain amount of biomaterial and cells into the disc in order to be able to achieve a certain intensity of treatment. This is the reason why this study methodologically proceeded by injecting “as much as possible” exemplary biomaterial into the disc. This definition without quantification was chosen to determine the injectable volume and to reflect an approach that is as close to practice as possible. In subsequent in vitro experiments, it is now convenient to inject a defined amount in the range determined, 0.73 mL, into papain-treated bovine tail disc of level CY34, allowing for a high variability in the volume and cell concentrations in further studies.

However, when using bovine tail discs, limitations must also be taken into account when it comes to transferability to the human spine. The tail discs have a different anatomy, no facet joints, and a different physiological loading condition as well as an altered matrix composition [19,23,78]. In contrast, they are characterized by good homogeneity, little ethical concerns, easy availability, and their biological and biomechanical properties have been extensively investigated. At the same time, the aim of this study was to further understand and improve the potential of the bovine tail model.

Perhaps deeper insights could have been gained by testing lower concentrations of papain, such as by Chan et al., where cavities for 3 or 15 U/mL of papain were not created and some minor proteoglycan staining was visible in the Safranin-O sections for 3 U/mL papain [29]. However, the aim of the study was to create a visible cavity as fast as possible (in 7 days). As previously mentioned, the papain concentration of 65 U/mL used in the present study was substantially less than those reported in other biomechanical investigations in which chymopapain was used [39,40,41,49,53,79]. Furthermore, comparisons are also difficult, as publications state the amount of papain differently (enzyme weight, vials, units, volume), and the weighed enzyme amounts have different activities depending on the batch [80]. So, we recommend indicating the enzyme amount in units per volume and the volume that is injected.

In this new in vitro model, neither the duration of the papain enzyme’s activity nor the possibility of papain stopping has been investigated. Potter reviewed in 1961 how papain was distributed in rabbits in vivo and that alpha-2-macroglobulins from the inflowing extracellular fluid could inactivate the free enzymes [79]. This limits the comparability of papain digestion in vivo vs. in vitro and highlights the importance of the present study. It is likely that the swelling conditions, cyclic loading, and the exchange of the culture medium also have an influence, since, in vivo, the living organism and blood flow play a role in how proteoglycan components move from the disc through the blood for excretion by the kidney [79]. This also explains why lower amounts of papain in vitro compared to in vivo are sufficient to reliably produce cavities in bovine tail IVDs. In addition, it is likely that cavities are less prevalent in vivo because, as described above, the organism probably counteracts against the enzyme and so other amounts of enzyme have been used in clinical practice [79]. Another consequence to be drawn from Potter represented the active residues of papain which could still be present in the model after seven days and during subsequent testing of the biomaterials. Hence, the inactivation of papain should be considered in the later application, since progressive changes are still observable after 7 days [29,80].

Although studies have shown that papain digestion can mimic different degrees of disc degeneration well in terms of disc height, water content, or even T2 relaxation time patterns [27,28,29,81], future studies should investigate how the biomechanical and structural effects of disc degeneration can be better modeled in vitro. In particular, with the papain protocol used here, the significant ROM increase does not correspond well to the biomechanical effects of physiological degeneration where a decrease in ROM is typically observed [82,83,84]. However, we have observed findings that may be helpful for additional new models. Complex loading produced defects like fissures and clefts independently of the enzyme digestion (Figure 5c), which could help to improve existing degenerative models. We think that cracks and fissures would be very beneficial for testing biomaterials in the disc, e.g., in order to observe how a novel biomaterial would distribute into these gaps, and this has not been carried out so far [27,85,86]. Furthermore, it can be hypothesized that the combination of lower concentrations of papain (just affecting the NP) and complex loading could be a promising solution to simulate more degenerative changes in the bovine tail disc than simulating nucleotomy with cavities. Complex loading would simulate the fissures and clefts, whereas a small dose of papain would change matrix composition and reduce intradiscal pressure for injections. This idea is also supported by the fact that there are already IVD culture models with degenerative loads and that these fissures, which are caused by complex loading, also correspond to the natural development of IVD degeneration, in which mechanical loads may also play a role [55,87]. Another prospective investigation could be cell viability which we did not measure in the present study and where no direct conclusions can be drawn. Cell viability is an important characteristic of organ culture models and would open more advanced experiments that investigate the interplay of cells, biomaterials, segmental flexibility, as well as dynamic loading. However, previous measurements using similar experimental protocols provide some answers to this question. For example, it has been described that the cavity itself no longer contains any tissue and therefore no cells [28], but that the cell viability in the remaining tissue after papain treatment (5–150 U/mL) can be reported to be at 100% 7 days after papain injection as well as at 68% to 82% 10 days after papain injection [27,29]. Cartilaginous endplates show cartilage cells of normal appearance [49]. Even though the bovine tail is a widely accepted in vitro model in disc research, in the future, it can be hypothesized that this organ culture approach can also be transferred to other animal models with fresh functional spinal units from species such as dog, sheep, and goat, since it is already known from dogs, humans, rabbits, and several other species that papain acts enzymatically [19]. All these models have no ethical concerns when the animals are slaughtered for alimentary purposes and thus reduce animal suffering and are easily available.

## 3. Conclusions

While the biological effects, such as gene expression, of chemonucleolysis with papain on bovine intervertebral discs have been extensively studied, the study provides further insight into the biomechanical effects on ROM, NZ, and disc height in relation to histology and the glycosaminoglycan content. This was possible by a new experimental approach that digests the discs with papain for seven days, while allowing them to be clamped into testing machines for biomechanical testing. By using fresh tissue and organ culture, the specimen can be used longer for testing, as no deterioration or degradation was observed. Thus, the enzyme papain has more time to digest the proteoglycans. The formation of cavities by a high concentration of papain leads to an increase in the ROM and NZ and a reduction in the disc height, mimicking the biomechanical effects of nucleotomies and disc herniations. Therefore, papain-digested discs are likely to simulate pathologies such as partial discectomies, nucleotomies, or herniations in biomechanical experiments in vitro. In particular, the intact anulus provides new possibilities for in vitro studies compared to the classical nucleotomy using surgical tools and a subsequent anulus defect. Papain also has a pronounced effect on the anulus fibrosus and leads to a strong biomechanical weakening in the axial rotation plane. After applying the protocol used in the present study, a median volume of 0.73 mL biomaterial can be injected into the bovine tail discs of level CY34, and subsequently biomechanical testing can be performed. This new model now allows for the preclinical testing of gels injected into the IVD.

## 4. Materials and Methods

### 4.1. Specimens

**Figure 6:**
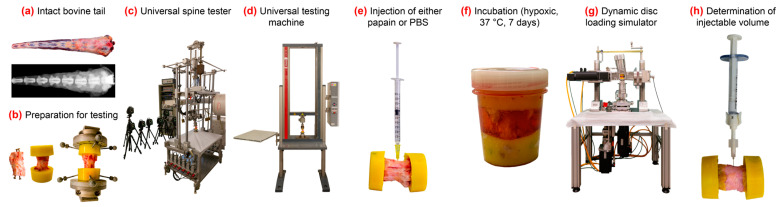
Method and machines. (**a**) Fresh bovine tail segment intact with antero-posterior X-ray. (**b**) Preparation and embedding of mono-segmental specimen with flanges and motion tracking markers. (**c**) Universal spine tester (Wilke et al., 1994 [88]) used for testing ROM and NZ with pure moments in three motion planes. (**d**) Universal testing machine for compression test to measure disc height change. (**e**) Injection of either papain or PBS as sham under sterile conditions. (**f**) Incubation in cell culture medium in hypoxic environment at 37 °C. (**g**) Dynamic disc loading simulator (Wilke et al., 2016 [89]) for applying complex loading for 2700 cycles. (**h**) Double chamber syringe for injecting radiopaque hydrogel for determining the injectable volume.

In total, 40 mono-segmental specimens of level CY34 were collected from 40 bovine tails (12–24 months old), which were directly obtained after slaughtering (Ulmer Fleisch GmbH, Ulm, Germany). No ethical approval was required. Prior to preparation on the same day, the specimens were screened for signs of anatomic anomalies, growth abnormalities in the vertebral bodies, previous vertebral fractures, and degenerative changes using an anterior–posterior X-ray (DR Panel PIXX1417, PIXXGEN Corporation, Anyang-si, Gyeonggi-do, Republic of Korea) (Figure 6a). The size of the IVDs was determined using X-ray images in the anterior–posterior and latero-lateral planes (ImageJ 1.53v), and the mean diameter and mean height were calculated. The segments were blindly assigned to a papain-treated group (n = 21) and a sham-treated group with PBS (n = 15). Two additional specimens were used to visualize the fluid distribution within the disc directly after injection and 24 h after injection in culture medium (n = 2). Another specimen was used to visualize the cavity under X-ray with contrast agent (n = 1), and one specimen served as a histological reference to compare the effects of the complex loading on the tissue integrity (n = 1). For biomechanical testing, the flanges were screwed on the hardened PMMA blocks of each functional spinal unit (Figure 6b).

### 4.2. Enzymatic Digestion

Crystallized papain (P4762, Sigma-Aldrich, St. Louis, MO, USA) was dissolved and stored in 50 mM sodium acetate buffer (Sigma-Aldrich), with a ratio of 650 units papain per 1 mL according to the manufacturer. This stock solution was mixed shortly prior to the injection with a PBS buffer (14190250, Gibco, Carlsbad, CA, USA) of pH 6.4 containing 5 mM ethylenediaminetetraacetic acid (EDTA, Sigma-Aldrich) and 10 mM L-cysteine (Sigma-Aldrich), resulting in a final papain concentration of 65 U/mL. After cleaning and disinfecting the functional spinal unit with saline containing 20% penicillin/streptomycin (10,000 U/mL–10 mg/mL, Gibco) and 10% amphotericin B (250 μg/mL, Sigma-Aldrich), the following steps were performed under sterile conditions. First, 200 µL of papain solution was injected into the center of the discs of the papain group with a needle size of 30 G (Figure 6e). Next, 200 µL of PBS was injected into the sham group discs. After injection, the motion segments of both groups were incubated under a free-swelling condition at 6% O_2_ and 37 °C for seven days using 60 mL high-glucose Dulbecco’s Modified Eagle Medium (Gibco) per specimen, supplemented with 5% fetal bovine serum (Sigma-Aldrich), 1% penicillin/streptomycin, 0.5% amphotericin B, 1% non-essential amino acids (Gibco), and 1.5% 5 mol/L NaCl/0.4 mol/L KCl solution to adjust the osmolarity of the medium to 400 mOsm. On day three, the cell culture medium was exchanged. Incubation was realized using sterile container with a not fully closed screw cap (100 mL, Polyethylene, 75.562.105, Sarstedt AG & Co. KG, Nümbrecht, Germany) (Figure 6f).

### 4.3. Injections, X-ray Control, and Macroscopic Views

All the specimens of both groups were imaged in the intact state, after incubation, and after loading in the antero-posterior and latero-lateral planes (DR Panel PIXX1417, PIXXGEN Corporation, Gyeonggi-do, Republic of Korea). In order to understand the injection process, two exemplary discs were injected with a contrast agent (Imeron 400, Bracco Imaging Deutschland GmbH, Konstanz, Germany) and incubated in the cell culture medium (n = 2). The status of the contrast agent was analyzed with an X-ray (FCR Prima 20 VET, Fujifilm Holdings Corp., Tokyo, Japan) directly after injection and 24 h after injection. A total of four discs for the papain and sham groups were dissected and photographed transversally on day 7 after complex loading. It was also noted whether cavities had occurred and how they appeared morphologically. For representative discs of both groups, papain and sham, the injectable volume was determined using a hyaluronan-based hydrogel (TETEC AG, Reutlingen, Germany) (n = 6) (Figure 6h). After incubation and complex loading, hydrogel was injected until an abrupt resistance was felt on the piston during injection and the disc started to bulge. The volume was recorded on the syringe scale.

### 4.4. Flexibility Tests

ROM was measured under quasi-static conditions in three subsequently tested anatomic planes, FE, LB, and AR, according to the widely approved testing standards with a universal spine tester (Figure 6c) [58,88]. Next, 3.5 cycles of pure moments of ±1 Nm were applied with a speed of 1.0°/s without preload. Moments of ±1 Nm were sufficiently high to achieve a progressive increase in stiffness, but low enough to avoid structural damage to the specimens during testing and to avoid reaching the maximum conditions of the testing machine due to the high flexibility of the bovine tails. Motions in terms of ROM and NZ were determined using a motion tracking system (Vicon Nexus 1.4.116, Vicon Motion Systems Ltd., Oxford, UK) with six cameras (Type MX13, Vicon Motion Systems Ltd., Oxford, UK). Flexibility was measured in the intact state, after incubation on day 7, and after loading on day 7. For every motion plane, the total ROM and total NZ were calculated as the sum of the ranges of both sides.

### 4.5. Disc Height

The disc height change was measured with a low compression force in a universal testing machine (Z01, Zwick Roell, Ulm, Germany) at a defined loading plateau of 30 N (Figure 6d). The value of 30 N and the method was derived from a previous study with human segments using 100 N by adapting the area ratios of human discs to the bovine discs [74,78]. The disc height was measured intact, after incubation on day 7, and after loading at day 7. The change in disc height was defined as a difference between the initially intact measured value and the value measured at all subsequent time points.

### 4.6. Complex Loading

All specimens of both groups were exposed to complex loading using the same parameters in order to compensate disc swelling from incubation. A custom-built dynamic disc-loading simulator applied cyclic axial compression of 50–450 N at 3 Hz [89], which was simultaneously superimposed by a rotation in FE and left–right LB (both ±10°) acting as out-of-phase sine and cosine waves at 1 Hz (Figure 6g) [90]. After incubation, loading was applied for 1 h, corresponding to 2700 cycles at 1 Hz and containing short intervals for re-adjusting the compression force baseline to 150 N. The magnitude of the complex loading after incubation in the cell culture medium was chosen in such a way that the stiffening mechanical behavior by fluid uptake was compensated and the intact state was reached as close as possible. This was developed in the pretests.

### 4.7. Histology and Proteoglycan Content Quantification

Six discs per group, papain and sham, as well as one additional disc without dynamic loading and without papain treatment were shock-frozen after the dissection and removal of the endplates. Then, after a careful thaw, the discs were cut into halves axially; one half was processed for histology and the other one for proteoglycan quantification [91]. Following fixation in formalin for 48 h, the entire disc halves were washed under running tap water for 2 h, dehydrated with an ascending series of alcohol, and embedded in paraffin wax. Tissue sections with 7 µm thickness were collected, deparaffinized in xylene solution, and rehydrated through a graded series of ethanol. Safranin-O/Fast Green staining with 0.1% Safranin-O (Chroma Gesellschaft, Münster, Germany) and 0.02% Fast Green (Fluka, Buchs, Switzerland) was performed to identify proteoglycan distribution. The sections were imaged using a light table (White Screen Spezialleuchten GmbH, Weilheim, Germany) and a DSLR (Nikon D7100, Nikon Corporation, Tokyo, Japan). Sections were additionally imaged with brightfield microscopy (DMI6000 B, Leica Microsystems GmbH, Wetzlar, Germany). Punches (six in AF and six or less in NP depending on the cavity) were taken from the other half of the disc. After mincing the punches, the tissues were freeze-dried and digested overnight at 56 °C using 0.5 mg/mL proteinase K (P6556, Sigma-Aldrich). Sulphated glycosaminoglycan content was determined using the Blyscan assay kit (B1000, Biocolor, Carrickfergus, UK). The glycosaminoglycan content was normalized to the dry weight of the tissue.

### 4.8. Data Analysis and Statistics

Data were collected and processed using Microsoft Excel 16.87 2024 (Microsoft Corp., Redmond, WA, USA) and then statistically analyzed using IBM^®^ SPSS^®^ Statistics Version 29 (IBM Corp., Armonk, NY, USA). The Shapiro–Wilk test was used for determining the normality assumption of all data. For parametric analysis, a paired *t*-test was used to analyze the differences between the papain and sham groups, and an unpaired *t*-test was used to analyze differences between the different time points. Cohen’s term d was calculated to estimate the effect size of the *t*-tests. For non-parametric analysis, the Mann–Whitney U test was conducted to calculate differences between the groups (papain vs. sham) and the Friedman test with Dunn–Bonferroni post hoc was performed to determine the differences between the time points. All tests were performed with a significant level set to α = 0.05.

## Data Availability

All data are stored at the Institute of Orthopaedic Research and Biomechanics, Centre of Trauma Research Ulm, Ulm University, Germany.
